# Supplementary figures and images for: Progranulin A Promotes Compensatory Hepatocyte Proliferation via HGF/c-Met Signaling after Partial Hepatectomy in Zebrafish
Source: Int J Mol Sci. 2021 Oct 18;22(20):11217. doi: 10.3390/ijms222011217 (PMC8538350; doi:10.3390/ijms222011217)

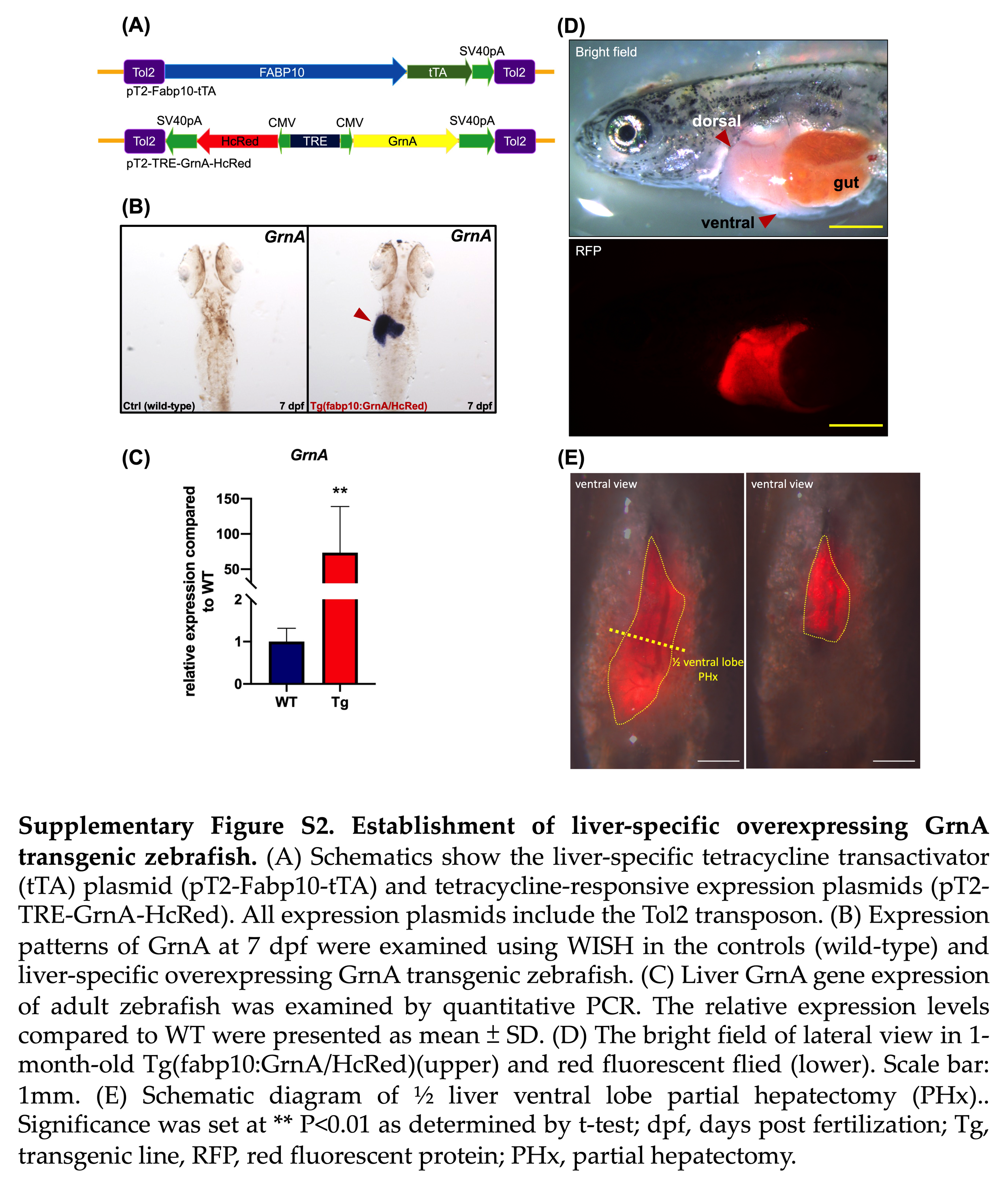

Supplement: Supplementary file 1 [file ijms-22-11217-s001.zip › supplementary material/Supplementary Figure S2-final version.tiff]

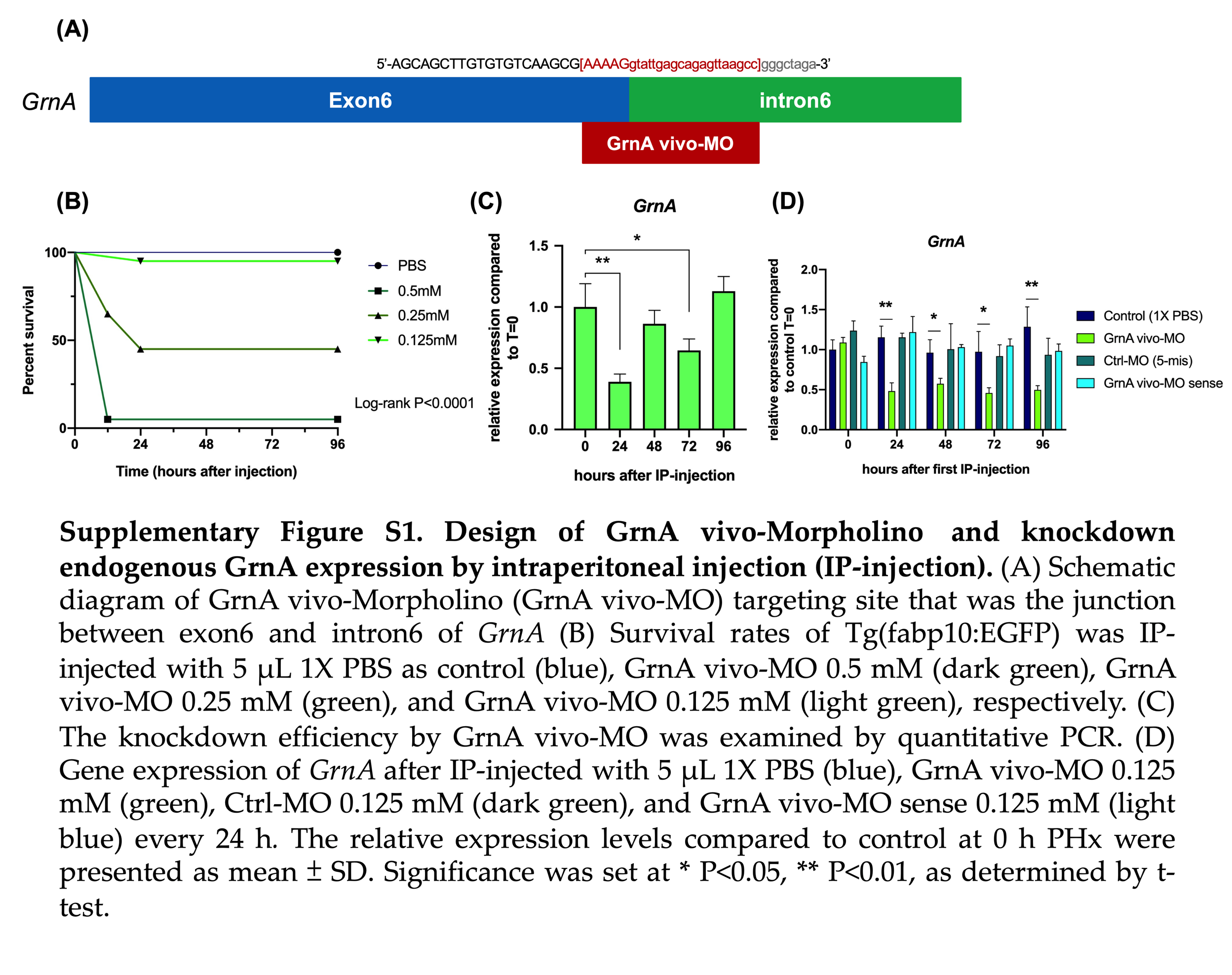

Supplement: Supplementary file 1 [file ijms-22-11217-s001.zip › supplementary material/Supplementary Figure S1-final version.tiff]
